# Supplementary material for: Near-Infrared Light Irradiation of Porphyrin-Modified Gold Nanoparticles Promotes Cancer-Cell-Specific Cytotoxicity
Source: Molecules. 2022 Feb 12;27(4):1238. doi: 10.3390/molecules27041238 (PMC8879323; doi:10.3390/molecules27041238)
Supplement: Supplementary file 1 [file molecules-27-01238-s001.zip › molecules-1559227-supplementary.pdf]

Supporting Information

Article

Near-Infrared Light Irradiation of Porphyrin Modified Gold Nanoparticles Promotes Cancer Cell Specific Cytotoxicity

Hiromi Kurokawa [1,2,*], Atsushi Taninaka [3,4], Toru Yoshitomi [5], Hidemi Shigekawa [3] and Hirofumi Matsui [1]

1 Faculty of Medicine, University of Tsukuba, Ibaraki 305-8573, Japan; hmatsui@md.tsukuba.ac.jp
2 MoBiol Technologies Corporation, Ibaraki 305-0031, Japan
3 Faculty of Pure and Applied Sciences, University of Tsukuba, Ibaraki 305-8573, Japan; jun_t@bk.tsukuba.ac.jp (A.T.); hidemi@bk.tsukuba.ac.jp (H.S.)
4 TAKANO Co., Ltd., Nagano 399-4301, Japan
5 Research Center for Functional Materials, National Institute for Materials Science, Ibaraki 305-0044, Japan; YOSHITOMI.Toru@nims.go.jp (T.Y.)
* Correspondence: hkurokawa.tt@md.tsukuba.ac.jp

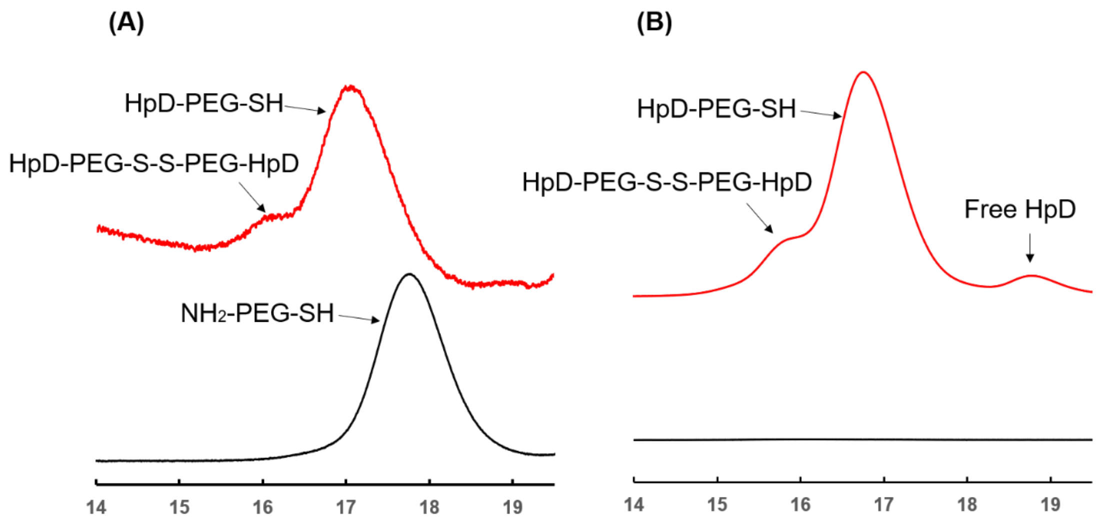

Figure S1. Gel permeation chromatograms of (black line) NH_2-PEG-SH and (red line) HpD-PEG-SH, which were measured by high-performance chromatography connected to a DP-8020 pump (TOSOH, Japan), a CO-8020 column oven (TOSOH, Japan), a UV-8020 ultraviolet detector (TOSOH, Japan), and an RI-2031 refractive index detector (JASCO, Japan) with Shodex OHpak SB-803HQ columns (Showa Denko, Tokyo, Japan). DMF containing 10 mM LiCl was used as the eluent at a flow rate of 0.5 mL/min at 40 °C. (**A**) The refractive index chromatograms of (black) NH_2-PEG-SH and (red) HpD-PEG-SH were shown. (**B**) The ultraviolet chromatograms at 400 nm of (black) NH_2-PEG-SH and (red) HpD-PEG-SH were shown.

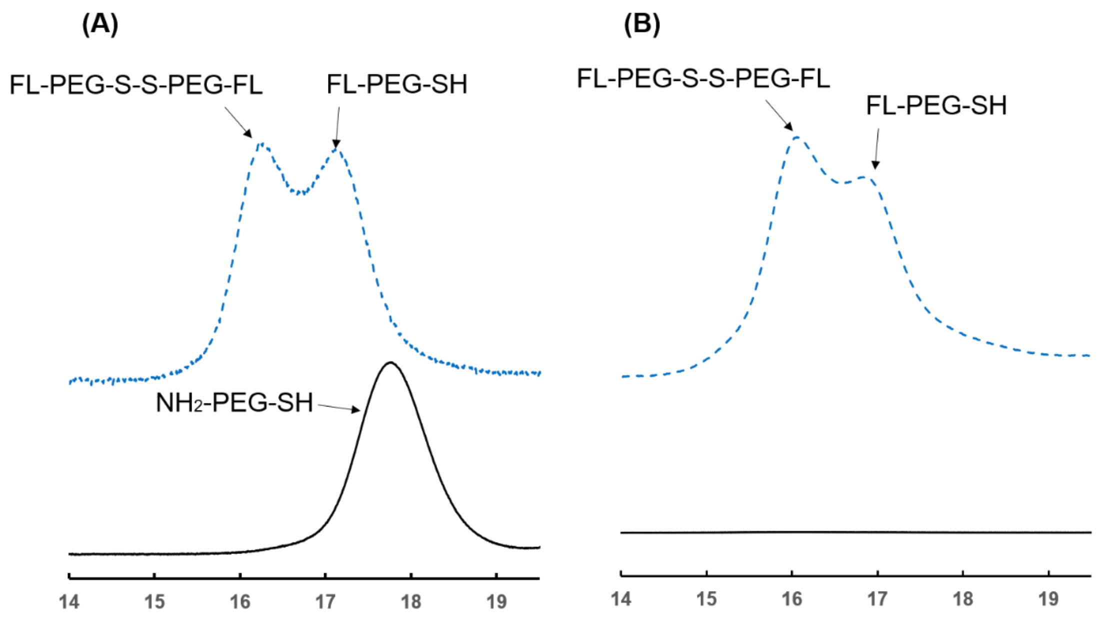

Figure S2. Gel permeation chromatograms of (black line) NH_2-PEG-SH and (blue dot line) FL-PEG-SH, which were measured by high-performance chromatography connected to a DP-8020 pump (TOSOH, Japan), a CO-8020 column oven (TOSOH, Japan), a UV-8020 ultraviolet detector at 480 nm (TOSOH, Japan), and an RI-2031 refractive index detector (JASCO, Japan) with Shodex OHpak SB-803HQ columns (Showa Denko, Tokyo, Japan). DMF containing 10 mM LiCl was used as the eluent at a flow rate of 0.5 mL/min at 40°C. (**A**) The refractive index chromatograms of (black) NH_2-PEG-SH and (blue) FL-PEG-SH were shown. (**B**) The ultraviolet chromatograms at 400 nm of (black) NH_2-PEG-SH and (blue) FL-PEG-SH were shown.

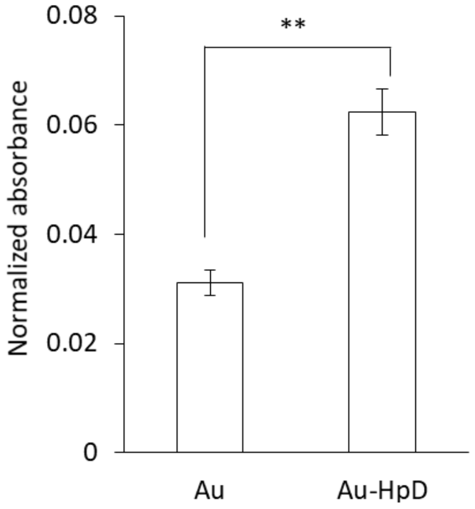

Figure S3. Intracellular AuNPs and Au-HpD accumulation in cancer cells. Data are expressed as means ± SD (n = 5). ** $p < 0.01$. RGK1 cells were seeded in 12-well plates at a density of 5×10^4 cells/well and incubated at 37 °C for 24 h. AuNPs or Au-HpD was added to cells and 24 h after cells were rinsed with phosphate buffer solution (PBS), and lysed in 100 µL of RIPA buffer. The cell homogenates were transferred to a 96-well plate and the absorbance was measured at 870 nm using a Synergy H1 microplate reader (BioTek Instruments Inc., Winooski, VT, USA).
